# Infant feeding counselling in Uganda in a changing environment with focus on the general population and HIV-positive mothers - a mixed method approach

**DOI:** 10.1186/1472-6963-10-260

**Published:** 2010-09-06

**Authors:** Lars T Fadnes, Ingunn Marie S Engebretsen, Karen Marie Moland, Jolly Nankunda, James K Tumwine, Thorkild Tylleskär

**Affiliations:** 1 Centre for International Health, University of Bergen, Bergen, Norway; 2 Faculty of Health and Social Sciences, Bergen University College, Bergen, Norway; 3 Department of Paediatrics and Child Health, Makerere University, Kampala, Uganda

## Abstract

**Background:**

Health workers' counselling practices are essential to improve infant feeding practices. This paper will assess how infant feeding counselling was done and experienced by counsellors and mothers in Eastern Uganda in the context of previous guidelines. This has implications for implementation of the new infant feeding guidelines from 2009.

**Methods:**

This paper combines qualitative and quantitative data from Mbale District in Eastern Uganda. Data was collected from 2003 to 2005 in a mixed methods approach. This includes: key-informant interviews among eighteen health workers in the public hospital, health clinics and non-governmental organisations working with people living with HIV, fifteen focus group discussions in the general population and among clients from an HIV clinic, two cross-sectional surveys including 727 mothers from the general population and 235 HIV-positive mothers.

**Results:**

The counselling sessions were often improvised. Health workers frequently had pragmatic approaches to infant feeding as many clients struggled with poverty, stigma and non-disclosure of HIV. The feasibility of the infant feeding recommendations was perceived as challenging among health workers, both for HIV-positive mothers and in the general population. Group counselling with large groups was common in the public health service. Some extra infant feeding teaching capacities were mobilised for care-takers of undernourished children. A tendency to simplify messages giving one-sided information was seen. Different health workers presented contradicting simplified perspectives in some cases. Outdated training was a common concern with many health workers not being given courses or seminars on infant feeding since professional graduation. Other problems were minimal staffing, lack of resources, and programs being started and subsequently stopped abruptly. Many of the HIV-counsellors in the non-governmental organisations got extended training in counselling which seemed to be beneficial.

**Conclusions:**

Health workers were faced with challenges related to workload, resources, scientific updating, and also a need to adjust to frequent changes in programs, recommendations and guidelines. The clients were faced with difficult choices, poverty, lack of education and stigma. Feasibility of the recommendations was a major concern. Systematic approaches to update health workers should be a priority.

## Background

Effective and feasible interventions that reduce child mortality include promotion of immediate and exclusive breastfeeding for six months, improved complementary feeding, and micronutrient supplementation with zinc and vitamin A [1-7]. Breastfeeding has been raised on the agenda of World Health Organisation (WHO) and UNICEF during the last two decades with initiatives as the Baby-Friendly Hospital/Health Initiative (BFHI) launched in 1991, and the Integrated Management of Childhood Illness (IMCI) launched in 1995 [8,9]. Many sub-Saharan countries adapted these programmes, and Uganda was one of the pilot countries for the IMCI-initiative [10]. Even though the BFHI and IMCI-initiatives strived towards promoting, protecting and supporting exclusive breastfeeding, numerous reports have shown widespread and prolonged breastfeeding, but limited practice of exclusive breastfeeding [11,12].

Transmission of HIV from mother-to-child through breastfeeding was identified in 1985 [13]. Since then, several reports have demonstrated confusion regarding infant feeding counselling among health workers in areas with a heavy burden of HIV [14,15]. This may have caused a spill-over effect hampering breastfeeding practices when recommendations aimed at HIV-positive mothers were adopted into the general population [16,17]. To reduce HIV-transmission, a prevention of mother-to-child transmission (PMTCT) programme was implemented in Uganda in 2000 [18]. The recommendations on infant feeding in the context of HIV changed several times with the growing knowledge, which was challenging to the counselling of infant feeding [19].

A number of studies have described improved HIV-free survival with exclusive breastfeeding compared to replacement feeding among infants of HIV-positive mothers [20-22]. For children infected with HIV, prolonged breastfeeding is associated with a better prognosis than a shorter duration of breastfeeding [23]. Still, an HIV-transmission risk exists with exclusive breastfeeding [20,21]. Recent studies have shown a reduction in HIV transmission by combining exclusive breastfeeding with antiretroviral prophylaxis [24-27]. This has led to a pivotal shift in the WHO guidelines on HIV and Infant Feeding from November 2009 [28-30]. The new guidelines are emphasising the importance of breastfeeding more than earlier, and recommend antiretroviral treatment to the mother when appropriate, and antiretroviral prophylaxis to the infant.

Sub-optimal infant feeding practices in Eastern Uganda has recently been shown, both in the general population and among HIV-positive mothers [11,31-33]. Health workers' counselling practices are key factors to improve the feeding practices [14]. How did the health workers' counselling practices influence infant feeding practices in this area? The aim of this paper is to assess how infant feeding counselling was done and experienced among counsellors and mothers in Eastern Uganda in the context of previous guidelines. This has implications for implementation of the new guidelines.

## Methods

The infant feeding counselling situation was assessed with a mixed-method approach including both qualitative and quantitative data from health workers and health clients in the same study catchment area of Mbale District, Eastern Uganda. The data collection period between 2003 and 2005 was a period of changes in infant feeding guidelines, and can therefore provide useful information on adaptations to changes in guidelines.

### Mixed methods approach

In line with the 'concurrent nested' design described by Cresswell et al [34], this paper combines both qualitative and quantitative data which have been carried out in parallel and integrated in the design, implementation and analysis phase. This study emphasises findings from the qualitative data which is triangulated with quantitative data. Detailed descriptions of the quantitative data packages exist elsewhere [11,31]. The multilevel designs framework which in this case involves key informant interviews for health workers and focus-group discussions for mothers has been well described by Tashakkori and Teddlie [35]. A list of the different data packages (DP) is presented below:

### Data packages in the study

DP 1 Thirteen key informant interviews with health workers from nine public sector institutions comprising nurses, midwives and clinical officers working with child health and infant feeding guidance were conducted in 2003 and 2005.

DP 2 Five key informant interviews among health personnel working with HIV positive people from a public hospital and in non-governmental organisations were carried out in 2005.

DP 3 Seven focus-group discussion among HIV-positive people were held at an HIV-clinic in 2005: 5 groups involving mothers below 35 years of age, one group with HIV-positive men, and one with HIV-positive women above 35 years of age.

DP 4 Eight community-based focus group discussions from 2003 highlighting the views of toddler parents regarding infant feeding practises (in press).

DP 5 A community-based cross-sectional survey was conducted in 2003 where 727 mothers were interviewed. This is analysed with regard to infant-feeding practices and growth determinants [11,32].

DP 6 A cross-sectional study was conduced in 2005 where 235 HIV-positive mothers-child pairs recruited from an HIV-clinic were interviewed. This is analysed with respect to feeding practices and predictors of feeding behaviour [31,36].

Some findings have been published from data package 4, 5 and 6, but this paper will focus on hitherto unpublished findings. The data packages will hence be referred to with superscript ^[DP]^.

### Study setting

Mbale has a population of 403,100 inhabitants [37]. The district is predominantly rural which is reflected in a 59% proportion of home deliveries (2008), but an antenatal attendance of 95%. The infant mortality rate is 76 per 1000 live births. The regional HIV-prevalence among fertile women was 6.2% (2004-5) [38]. Nearly all mothers practiced breastfeeding [11]. The Ugandan health system is characterised by a hierarchical structure, with a national referral hospital at the top level, followed by a regional hospital, health centre IV, health centre III at sub-country level and health centre II at the parish/community level. In Mbale District there was one regional hospital, one district hospital, and the majority of the sub-counties had a health centre III. Most women lived within 1-2 hours walking distance to a health centre II or III.

### Context of health education

Health education sessions were conducted in all public health facilities that were included in this study ^[DP1,2]^. The sessions usually lasted for 30-60 minutes. The topics being taught in the public health services and the way the sessions were organized varied. Feeding of infants was one of many themes that were covered during health education sessions. Other common themes were care during pregnancy, nutrition of pregnant women, postnatal care, sexually transmitted diseases including HIV, family planning, prevention of malaria, use of medicines, hygiene, first aid of conditions like diarrhoea with oral rehydration, and immunization of babies. The number of clients attending health education in the public health facilities ranged from around 10-200 per day, typically involving 20-50 mothers in each health education session. In the non-governmental organisations, individual counselling had a stronger stand. Regarding HIV, the public health services were moving towards the 'provider-initiated routine HIV counselling and testing algorithm' [39].

### Interviews

Key informant interviews were conducted with health personnel and 18 key informants were purposely selected. The main selection criterion was that the health workers participated in health education. The participants were recruited by approaching most public health facilities within Mbale Municipality and surroundings which could be reached within a one hour minibus drive. The informants comprised men and women within the age range 20-60 years. The mean duration of the interviews was one hour. The two first authors conducted the interviews with the health workers in English, tape-recorded and transcribed them. The first author was responsible for data package 2, 3, and 6, and the second author for 1, 4 and 5.

Mothers from the same area were interviewed in focus groups containing 5-11 participants and lasting about one hour. The focus groups were moderated by trained research assistants under supervision of the two first authors, and were conducted in the local language Lumasaaba, tape recorded, translated and transcribed. In the quantitative data packages, structured interviews were performed by data collectors.

### Analysis

Inductive thematic content analysis was chosen [40-42]. Systematic reading, coding and re-categorisation were performed by the two first authors. After identification of themes, inter-consistency checks were done. The following main themes emerged: 1) *The context influencing infant feeding teaching*; 2) *Feeding of severely ill children*; 3) *Challenges related to breastfeeding; *4) *The counselling process; *5) *Simplifying messages; *6) *HIV-infected mothers' disclosure and choice*; 7) *Feasibility and capacity challenges*. These themes will be mirrored from the different data packages, and are summarized in table S1 (additional file 1).

### Ethics

All participants signed informed consent. Ethical approval was obtained from Makerere University, Faculty of Medicine Ethics and Research Committee, and the Uganda National Council for Science and Technology.

## Results and discussion

### The context influencing infant feeding teaching

Although systematic approaches were described in some clinics, teaching in the public health system often appeared to be improvised according to the perceived needs of the clients, or the planned activities in the units ^[DP1,2]^. Most of the health workers in the public health services had their current knowledge on infant feeding guidelines from their training as professionals. Some of the health workers had attended specific workshops on infant feeding, including one who was trained within the IMCI framework of WHO. The infant feeding messages varied depending on whether the health workers had updated knowledge.

Infant feeding teaching and health education were regarded as important among health workers ^[DP1,2]^. In most cases the health educators got positive feedback as '*we have not known this.' *Women being busy with work who just came for treatment did pay less attention to the teaching, while those *'who come with malnourished children - they usually pay much attention.' *Great interest was expressed as motivating for the health workers. A factor opposing this enthusiasm was a heavy work load. '*The number of services is increasing, but we still have the same number of staff.'*

Regarding infant feeding, the health workers in the public services stressed the importance of breastfeeding and complementary feeding from six months. Terms like *'breastfeeding on demand' *and *'frequent breastfeeding' *were often referred to.

Many health workers had pragmatic approaches to infant feeding teaching, knowing that numerous mothers had challenges related to the feasibility of the infant feeding guidelines ^[DP1,2]^. As an example, women were taught how to dilute cow's milk and boil water for the baby when the health workers were informed that the mother had to leave the baby during daytime and could not manage exclusive breastfeeding.

The hygienic hazard of bottle feeding was often emphasised during the counselling sessions ^[DP1,2]^. The importance of hygiene in general and the hazard of some of the traditional practices were frequently underlined by the health workers. Many women and some men believed breasts had to be cleaned before breastfeeding, and told that this practice was encouraged by local health institutions ^[DP4]^. The health workers in contrast reported to encourage cleaning of the breasts only when the mothers had applied local medicines on the breasts.

The majority of the known HIV-positive mothers had discussed infant feeding with health personnel, 159 of 235 mothers (68%) ^[DP6]^. The counselling content that was reported most often was the question on when to introduce liquids and solids to infants. In the general population, the proportion reporting to have discussed infant feeding with health personnel was considerably lower, 107 of 727 mothers (15%) ^[DP5]^. The topic most often reported from the counselling sessions was breastfeeding technique. Some of the differences between the groups may be attributed to the fact that the recruited HIV-positive mothers received regular follow-up by health workers. Qualitative data from mothers in the general population revealed that mothers were informed about exclusive breastfeeding as a preferred practice, but they were equipped with limited strategies to maintain the practice ^[DP4]^.

### Feeding of severely ill children - an entry portal to infant feeding teaching

Although group teaching was dominating in the public services, they mobilised some extra teaching capacities to targeted individuals ^[DP1]^. Undernutrition was seen as an entry portal to discuss nutrition on an individual basis. 'S*ometimes when a mother comes with a child who is malnourished, we have to health-educate that mother.' *Harmful feeding practices were also discovered while treating children for other diseases. '*There are some who think that when a child is sick and just does not want to eat - they just stop there. For example if a child has measles, many people think that giving that child milk will worsen the condition. So when we talk with them about nutrition, food is part of the treatment.'*

Most health workers were referring children with severe undernutrition and clinical manifestations like kwashiorkor and marasmus on a monthly basis to the nutrition unit of the regional hospital. Referral itself was not necessarily the solution to undernutrition. *'You can tell the mother to go to the main hospital, and then she doesn't. She just goes back home. She doesn't have the money, and the (other) children are at home.' *The health workers related malnutrition to poverty, being sick with malaria, diarrhoea or cough, unawareness of nutritional values of food, poor hygiene, and the fact that young school girls often left their babies to grandmothers who did not manage substituting breast milk adequately. High birth rates were also considered to be partly responsible for the difficult situation many were facing. *'This malnutrition comes out of what? Poor family planning - you produce many (children), and cannot afford to feed them well and bring them up properly. So we also talk about family planning.' *Health workers struggled to counsel feasible alternatives for the infants: *'Most of these mothers don't have the food you ask them to give - some children come malnourished. Look at some kids - they are thin, they are wasted. Feeding down there is a very big problem.' *These problems seemed to affect many, and a study from the same area confirmed that numerous children were stunted and wasted [32]. This can have detrimental effects for the children in terms of increased morbidity and mortality [1].

### Challenges related to breastfeeding

Even if breastfeeding was regarded as the best infant feeding practice in the health institutions, some situations were reported where breastfeeding was debated, either among the mothers or among the health workers ^[All DP's]^. One such reason was new pregnancies. A pregnancy was a common reason to stop breastfeeding among mothers ^[DP4]^, but health workers on the contrary encouraged women to continue breastfeeding during new pregnancies ^[DP1,2]^. *'We are telling the mothers to breastfeed their children as much as possible and as long as possible.' *The belief that breastfeeding during pregnancies can be harmful has been reported from a study in Western Uganda [43]. That study described the belief that breastfeeding during pregnancy could cause kwashiorkor for the breastfed infant.

Both community members and health workers typified the group of busy mothers occupied with work and studies to often avoid breastfeeding ^[DP1-4]^. This was regarded as challenging for the health workers, but they had many pragmatic suggestions on how to overcome this challenge, such as having the baby brought to the mothers during working hours.

A challenge that many health workers faced, was the perceived lack of milk that some mothers experienced. In most cases the health workers would advise the mothers to have a varied diet and drink enough fluids to overcome the shortage of breast milk. They also acknowledged psychological stress as a factor contributing to breast feeding problems. Reasons why some mothers were suffering mentally were often related to economic worries and unstable family relations. The health workers showed great empathy for the extremely difficult situations many mothers bore when feeding their children.

Although a breastfeeding dilemma was observed in different situations, the HIV-positive mothers stood in the heart of this discussion. Avoiding breastfeeding was in many cases promoted as the ideal option for HIV-positive mothers from several of the health workers, both in the public health sector and among those working directly with HIV-positive mothers in the PMTCT programmes ^[DP1,2]^. Breastfeeding was thereby mainly regarded as an option for those who could not afford or manage replacement feeding. Many health workers acknowledged that their clients had economical constraints and therefore advised mothers to practice exclusive breastfeeding. When mothers had chosen a feeding option, some health workers highlighted the importance of sticking to the chosen option to avoid mixed feeding. When the HIV-positive mothers were asked what the health workers had told them related to breastfeeding, some reported that the transmission risk of breastfeeding was underlined, while other told that they were given advice on practical aspects of breastfeeding ^[DP3]^.

Mothers not breastfeeding were often suspected to be HIV-positive by their communities peers ^[DP2-4]^. Other illnesses like malaria and mental disorders were also held as likely explanations why mothers avoided breastfeeding. The choice to avoid breastfeeding was regarded as culturally highly controversial ^[DP3,4]^. Another view held by numerous young mothers as well as groups of men and older women, was that mothers not breastfeeding were immature and irresponsible. '*Some are mothers who lack responsibility and just want to enjoy their life*.'

There were mixed perceptions on exclusive breastfeeding. Among 235 HIV-positive mothers, 70% considered exclusive breastfeeding for several months to be harmful, while 30% considered it as beneficial ^[DP6]^. Water was regarded as a necessary addition to breastfeeding among 219 (93%) of the mothers, and exclusive breastfeeding was seen as insufficient for children ^[DP3,6]^. Regarding infant feeding practices, HIV-positive mothers breastfed shorter than mothers in the general population ^[DP5,6] ^[33]. Mixed feeding during the first half of infancy was widespread in both groups [11,31]. It seems like the message from the guidelines about the benefits of exclusive breastfeeding had not reached this population fully.

### The counselling process

Many of the health workers put much effort into communication and having a good, caring and empathic attitude ^[DP1,2]^. '*They have the potential, but they have not explored it. Our job is to open their minds*.' One-to-one counselling was a prioritised strategy in non-governmental organisations working with HIV-positive people, and often included teaching on infant feeding to parents when relevant. In order to ensure high quality of the counselling process, some health workers were concerned about not to have too many counselling sessions per day: *'if you counsel more than 10 people (each day), it is not effective counselling.'*

Some counsellors emphasised that they provided information to empower clients to take well informed choices ^[DP2]^. With this strategy, they stressed the importance of not making decisions for their clients. The clients themselves acknowledged this ^[DP3]^. *'We talked about very many things. So it was our own decision - we as individuals to decide what to do now.' *The topics discussed were often chosen in an interactive process between the clients and counsellors ^[DP2]^. '*So we allow them to tell their stories*.' Also some of the providers in the public sector used an interactive counselling strategy characterised by questions and answers ^[DP1]^.

Many of the HIV-counsellors in the non-governmental organisations got extended training in counselling ^[DP2]^. This type of training in communication skills seemed to improve teaching and counselling and could be beneficial also for an extended range of health workers conducting infant feeding teaching in the public sector.

### Simplifying messages

A tendency to simplify messages and exaggerate some perspectives at the expense of other perspectives during infant feeding counselling was observed ^[DP1,2]^. This may have been an effort to reach the audience, and was probably based on their interpretations of recent guidelines. An illustrating quote from a counsellor focusing on the hazards of breastfeeding was: '*When a mother is HIV-positive and breastfeeds her child, the child is likely to catch HIV*.' A similar simplistic message from a counsellor focusing on exclusive breastfeeding was: *'if you give any other feeds than breast milk, this means that the baby's alimentary canal will be infected*.'

This tendency of simplifying may have the advantage of not overwhelming the clients with complexity. Unfortunately it also carries the danger of confusing the listeners with incomplete information as many different health workers showed different parts of the picture. Some health workers were stressing the fact that HIV can be transmitted through breastfeeding, while others were focusing on the beneficial effect of exclusive breastfeeding. Clients who got contradictory simplistic messages may have had difficulties putting the pieces together to make informed choices. This way of informing clients in addition to varying routines and content of the infant feeding counselling, may have contributed to increase the amount of mixed feeding. Related issues have been pointed out in some Sub-Saharan countries. Chopra et al described counselling among health workers in Botswana, Kenya, Malawi and Uganda, and concluded that many overestimated the risk of HIV-transmission [19]. Similarly, Doherty et al described confusion among health workers in South Africa resulting in mixed feeding [14].

### HIV-infected mothers' disclosure and choice

Disclosure of HIV-status was a challenge to optimal feeding in many cases ^[DP1,2,3]^. In some cases, mothers breastfed their children when seen by others, while they were avoiding breastfeeding when being alone. *'I'm sorry, I can't (give replacement feeds) because the man will know (my HIV status), and he will send me away and will not even buy milk for this child. How am I then going to care for this child?' *Similarly, some HIV-positive men not having disclosed their status asked their wives not to breastfeed. This resulted in replacement feeding when the mother was seen by the husband, but breastfeeding when the husband was absent. The least fortunate infant feeding practice, mixed feeding during the first half of infancy, was thus often a consequence of not disclosing HIV-status. Not disclosing HIV-status has been reported to increase mixed feeding also in two South African studies [14,44]. However, the quantitative data did not show any significant differences in infant feeding practices between mothers having disclosed their HIV-positive status in their respective community compared to those who had not disclosed ^[DP6]^.

Disclosure of HIV may not be an easy choice as some women may have to pay a high price for disclosing their HIV status. Several of the HIV-positive people who disclosed their status told stories of being excluded from the family, being treated badly, losing their property or work, and being sent away from their homes ^[DP3,6]^. On the other hand, many who disclosed their HIV-status reported that they had been encouraged and supported by their communities. Some health workers reported a trend of improvement regarding stigma: '*Initially it was stigma, but stigma is dying*.' Studies from South Africa suggest that the mothers experiencing negative outcomes from disclosure are the exceptions rather than the rule [44-47]. Many report more support from family and friends when disclosing their HIV status. The weight of the stigma burden may be related to how well each setting has adapted to HIV, which in many settings seems to require some maturation time. As numerous settings still struggle with these adaptations, many HIV-positive people still face stigma as a heavy burden.

### Feasibility and capacity challenges

Health workers also brought up program changes as a factor that complicated their work ^[DP2]^. One example was a program supplying infant formula to HIV-positive mothers before it suddenly stopped. The mothers then asked the health workers what to do as they could not afford to buy replacement food. '*I don't have flour to make porridge. I can't afford milk*.' The counsellors found this hard to handle. '*So you find that the food the mother have at home is really not opted for the child - so you really fail to understand how to help them out of their situation*.' It has also been described that replacement feeding is opposing the view of motherhood, which can partly explain that it is often perceived as an unacceptable alternative [48].

In addition to acknowledging the mothers' challenges, many health workers in the public health sector expressed a deep concern for the situation of their institutions ^[DP1]^. Major concerns which made daily performances difficult were inadequate facilities including lack of separate rooms for deliveries, absence of soap, clean water, electricity, and drugs in stock. This was reported to be most precarious in the remotest areas. Inadequate facilities are a burden to the health workers and may impede counselling. These obstacles came in addition to their own perceived needs for refreshing courses, workshops and teaching material. A few of the health workers expressed a need for demonstration kits to ease the infant feeding sessions. The need for more staff was also pointed out by several health workers.

### Key principles discussed

This study has described the circumstances around infant feeding teaching and counselling in Mbale District, Eastern Uganda. It has highlighted variation in counselling and messages. Further, the study has highlighted some of the challenges and opportunities existing. Major challenges existed both in the health system and on the client level. Lack of resources, minimal staffing, inadequate training and follow-up, as well as meeting the clients challenges, were frequently reported from the health workers. The clients on the other hand were faced with poverty, difficult choices and stigma. Large counselling groups with ad hoc curriculum and irregular sessions might explain why mothers had heard about exclusive breastfeeding, but not integrated the messages. Health workers were concerned with feasibility of exclusive breastfeeding, and often suggested pragmatic solutions when they were faced with mothers who did not manage to practice the ideal options.

The economic situation of the public health services will unfortunately impede individualised counselling to the same extent as practiced in some non-governmental organisations. If more is invested in the public health system to upgrade the infant counselling services, it may be possible to improve the feeding situation [49]. A review on interventions to change health workers' behaviour identified workshops as one of the potent strategies [50]. Similarly, a Tanzanian and a Sudanese study trying to change health workers' practices showed substantial benefits of short seminars [51,52]. To maintain the knowledge, it was necessary to have follow-up or refreshing seminars. Based on the shortcomings observed in this study, it could be beneficial with short updating workshops for health workers on a regular basis to keep them up to date without taking too much valuable time. If different topics relevant for antenatal care could be integrated into e.g. annual workshops, important updates on guideline changes and changes in patient management could be provided. This could potentially streamline the health education and reduce the risk of confusion.

Counselling was given high priority in the HIV-positive population, but contradicting and simplified messages might have hampered some of the effects of the counselling on infant feeding. The new guidelines from 2009 advocate for '*informing mothers known to be HIV-infected about infant feeding alternatives*' [28]. Avoiding simplistic and diverging messages might be important to fulfil this objective. How the information is conveyed is likely to determine the choice of the mother. Many HIV-positive mothers will still face disclosure challenges in their respective communities. Without strategies in place to support them, it is difficult to avoid unfavourable feeding practices.

Experiences from this study can shed light on some aspects of implementation of the new guidelines on HIV and infant feeding [28]. These guidelines promote exclusive breastfeeding in contexts with a high HIV prevalence more than earlier guidelines did, and emphasise not to compromise breastfeeding in the general population. As implementation of guidelines and programs not always reach the aims that have been set, ongoing evaluation during implementation is essential [53,54]. There seems to be a need to promote, protect and support exclusive breastfeeding more; with increased emphasis in the public health system. Individualised and group peer-support of breastfeeding are promising strategies [55,56].

### Strengths and limitations

The strength of this study was the combined use of qualitative and quantitative data to shed light on the teaching, counselling and infant feeding situation in Eastern Uganda. This enabled a discussion of challenges related to choice of infant feeding and feasibility which is highly relevant to the new infant feeding guidelines in the context of HIV. There are inbuilt limitations with the design of the study: the integration of data was done post hoc and there are few descriptions on how this can be done as objective as possible [34]. For this reason, some of the discrepancies between messages from the health providers and the health clients observed while integrating the data, cannot be elaborated on. An example was the discrepancy reported on 'cleaning of breasts' from the health workers and the mothers. Some topics from the key-informant interviews could not be triangulated with quantitative data from the health clients. Still, the mixed triangulation approach enabled us to present and compare overlapping and diverging findings.

Regarding the qualitative data and the selection of informants, all interviewed health workers performed counselling or health education, but not all had it as their principal responsibility. Even though having tried to recruit informants from all relevant health providing institutions in the area, not all were reached. Traditional birth attendants may also have given counselling to some mothers. However, they were not included in this study. Although emphasising to the respondents that the information they gave could not be linked to them, respondents may have given socially desirable responses. Limitations of the quantitative data have been elaborated on elsewhere [33].

## Conclusions

This study showed that the quality of infant teaching and counselling might be compromised when adjusting to frequent changes in programs while experiencing a heavy work load. Major challenges for the health workers were lack of resources and minimal staffing, inadequate training and follow-up, as well as meeting the complicated situations of the clients. The clients were faced with difficult choices often associated with poverty, feasibility, stigma and disclosure of HIV.

With the new guidelines on infant feeding in the context of HIV from late 2009 being implemented, it is important not to repeat previous missteps. New guidelines need to be integrated in all relevant levels of the health system to reach out to the clients. The clients should experience a more streamlined education with confident health educators. Systematic approaches to update health workers should be a priority. More resources to the public health service in low-income country must be an aim.

Nevertheless, it is also important that future operational research evaluate acceptability and feasibility of infant feeding guidelines, and the effect of the recommendations on behavioural changes. Earlier experiences have taught us that research must go hand in hand with implementation of new guidelines. This could detect confusion among health personnel and health clients, and suggest early adaptations to increase acceptability and feasibility.

## Abbreviations

BFHI: baby-friendly hospital initiative; DP: data package; HIV: human immunodeficiency virus; IMCI: integrated management of childhood infections; PMTCT: Prevention of Mother-to-Child Transmission; WHO: World Health Organisation.

## Competing interests

The authors declare that they have no competing interests.

## Authors' contributions

LTF and IMSE: design, implementation, analysis and writing. JN and KMM: co-writing. TT and JKT: design and co-writing. All authors approved the final manuscript.

## Pre-publication history

The pre-publication history for this paper can be accessed here:

http://www.biomedcentral.com/1472-6963/10/260/prepub

## Supplementary Material

Additional file 1 Table S1: Summary of main findings from the different data packages sorted under themes [DP1-6]. Click here for file
