# Supplementary material for: Infant feeding counselling in Uganda in a changing environment with focus on the general population and HIV-positive mothers - a mixed method approach
Source: BMC Health Serv Res. 2010 Sep 6;10:260. doi: 10.1186/1472-6963-10-260 (PMC2944269; doi:10.1186/1472-6963-10-260)
Supplement: Additional file 1 — Table S1: Summary of main findings from the different data packages sorted under themes [DP1-6]. [file 1472-6963-10-260-S1.PDF]

Table S1: Summary of main findings from the different data packages sorted under themes [DP1-6]

	**Key informant interviews with health workers in**		**Focus group discussion among**		**Cross-sectional surveys**	
	**public health clinics and hospitals [DP1]**	**HIV-clinics and NGO's working with HIV [DP2]**	**HIV-positive people from an HIV-clinic [DP3]**	**General population (community-based) [DP4]**	**General population (community-based) [DP5]**	**HIV-positive people from an HIV-clinic [DP6]**
Context influencing infant feeding teaching	- improvised teaching<br>- training varied<br>- pragmatic approaches	- often have extended counselling training<br>- pragmatic approaches	- poverty was a challenge	- poverty was a challenge	- minority had discussed infant feeding with health worker	- majority had discussed infant feeding with health worker
Feeding of severely ill children	- some extra teaching capacities to targeted individuals<br>- poverty and knowledge reported as challenging	- poverty was a challenge	- poverty was a challenge	- poverty, social structure within the families and psychological difficulties were challenging	- poverty was a challenge<br>- clear association with infant feeding practices and socio-economic status	- poverty was a challenge<br>- clear association with infant feeding practices and socio-economic status
Challenges related to breastfeeding	- health workers had to work against traditional beliefs<br>- pragmatic options suggested for mothers not breastfeeding<br>- For HIV-positives, breast-feeding seen as 2. option	- health workers had to work against traditional beliefs<br>- busy mothers often avoided breastfeeding<br>- For HIV-positives, breast-feeding seen as 2. option	- HIV transmission during pregnancy often underlined<br>- busy mothers often avoided breastfeeding<br>- Mothers not breastfeeding were often suspected to be HIV-positive or accused of being irresponsible	- a pregnancy was a common reason to stop breastfeeding<br>- busy mothers often avoided breastfeeding<br>- Mothers not breastfeeding were often suspected to be HIV-positive or accused of being irresponsible	- exclusive breastfeeding often regarded as insufficient and practiced at a low rate<br>- mixed feeding common	- exclusive breastfeeding often regarded as insufficient and practiced at a low rate<br>- mixed feeding common

The counselling process	- variation in messages<br>- some used interactive counselling strategy	- Many health workers put much effort into communication and a good, caring and empathic attitude<br>- variation in messages	- positive feedback on health workers' counselling	- positive feedback on health workers' counselling		
Simplification of messages	- simplistic messages<br>- exaggerated some perspectives	- simplistic messages<br>- exaggerated some perspectives				
HIV-infected mothers' disclosure and choice	- HIV disclosure was a challenge	- HIV disclosure was a challenge<br>- less stigma<br>- mixed feeding seen as consequence of non-disclosure	- HIV disclosure was a challenge<br>- many pay high price for HIV disclosure<br>- many felt supported when disclosing HIV-status			- no differences in infant feeding practices seen between mothers having disclosed or not<br>- many pay high price for HIV disclosure
Feasibility and capacity challenges	- inadequate facilities, staffing & equipment<br>- need for training and follow-up, e.g. refreshing courses<br>- meeting their clients' complicated situations	- programs stopping abruptly<br>- meeting their clients' complicated situations	- difficult choices<br>- poverty<br>- stigma	- poverty<br>- difficult choices		- poverty<br>- stigma
